# Analysis of Chest-Compression Depth and Full Recoil in Two Infant Chest-Compression Techniques Performed by a Single Rescuer: Systematic Review and Meta-Analysis

**DOI:** 10.3390/ijerph17114018

**Published:** 2020-06-05

**Authors:** Chun-Yu Chang, Po-Chen Lin, Yung-Jiun Chien, Chien-Sheng Chen, Meng-Yu Wu

**Affiliations:** 1 School of Medicine, Tzu Chi University, Hualien 970, Taiwan; paulchang1231@gmail.com; 2 Department of Emergency Medicine, Taipei Tzu Chi Hospital, Buddhist Tzu Chi Medical Foundation, New Taipei 231, Taiwan; taipeitzuchier@gmail.com (P.-C.L.); holeyeye@yahoo.com.tw (C.-S.C.); 3 Department of Emergency Medicine, School of Medicine, Tzu Chi University, Hualien 970, Taiwan; 4 Department of Physical Medicine and Rehabilitation, Taipei Tzu Chi Hospital, Buddhist Tzu Chi Medical Foundation, New Taipei 231, Taiwan; jessica.kan.48@gmail.com

**Keywords:** cardiopulmonary resuscitation, two fingers, two thumbs, infant, chest compression

## Abstract

Pediatric cardiac arrest is associated with high mortality and permanent neurological injury. We aimed to compare the effects of the two-thumb (TT) and two-finger (TF) techniques in infant cardiopulmonary resuscitation (CPR) performed by a single rescuer. We searched PubMed, EMBASE, and CENTRAL for randomized control trials published before December 2019. Studies comparing the TT and TF techniques in infant CPR were included for meta-analysis. Relevant information was extracted for methodological assessment. Twelve studies were included. The TT technique was associated with deeper chest-compression depth (mean difference: 4.71 mm; 95% confidence interval: 3.61 to 5.81; *p* < 0.001) compared with the TF technique. The TF technique was better in terms of the proportion of complete chest recoil (mean difference: −11.73%; 95% confidence interval: −20.29 to −3.17; *p* = 0.007). CPR was performed on a manikin model, and the application of the results to real human beings may be limited. The TT technique was superior to the TF technique in terms of chest-compression depth, but with inferior chest full recoil. Future investigations should focus on modifying the conventional TT technique to generate greater compression depth and achieve complete chest recoil.

## 1. Introduction

Pediatric cardiac arrest is a challenge in emergency departments, with an estimated 16,000 pediatric cases annually reported in the United States, and it is associated with high mortality and permanent neurological injury [1,2,3]. In infants, demands of cerebral blood flow and energy metabolism are higher than those in adults due to neuronal maturation and synaptogenesis [3]. Early implementation of high-performance cardiopulmonary resuscitation (HP-CPR) increases survival rate and favorable neurological outcomes. The quality of cardiopulmonary resuscitation (CPR) depends on several factors, including chest-compression depth and complete chest recoil. On the basis of current concepts, it is recommended that infant CPR be performed with a chest-compression rate of 100–120 per minute, chest-compression depth at least greater than 40 mm, complete chest recoil, and minimal interruption of chest compression, while avoiding excessive ventilation. According to the recommendations of the 2015 American Heart Association (AHA) guidelines for infant CPR, two chest-compression techniques, the two-finger (TF) and two-thumb (TT) encircling chest compression, are recommended for infant resuscitation. The TF technique is recommended for a single rescuer, whereas the TT technique is recommended for two or more rescuers [4,5]. The TF technique is performed by placing two fingers on the lower third of the sternum for compression. In contrast, the TT technique involves two-thumb compression and thoracic squeeze, which theoretically provides more consistent compression depth, and higher arterial and coronary perfusion pressure than the TF technique does. However, there is a lack of strong evidence to confirm this hypothesis. In this meta-analysis, we compare chest-compression depth and full recoil between two infant chest-compression techniques in infant CPR performed by a single rescuer. 

## 2. Materials and Methods

### 2.1. Study Design

The study aimed to evaluate compression depth and full recoil between the TT and TF techniques during infant CPR. The study complied with recommendations made by the preferred reporting items for systematic review and meta-analysis (PRISMA) statement [6].

### 2.2. Search Strategy

Two authors (YJC and CYC) searched three major databases, namely, PubMed, EMBASE, and Cochrane Central Register of Controlled Trials (CENTRAL). Mesh terms from PubMed and CENTRAL, and Emtree terms from Embase were used in combination with the title/abstract tag (TIAB) or free-text words. The following terms were used to search for “CPR” using the “OR” operator: “cardiopulmonary resuscitation” OR “heart arrest” OR “heart massage” OR “chest compression”. The following terms were used to search for “infant” using the “OR” operator: “infant” OR “newborn” OR “neonate”. The following terms were used to search for “two-thumb and two-finger chest compression” using the “OR” operator: “two-thumb” OR “two-finger” OR “two-thumb chest compression” OR “two-finger chest compression” OR “infant-chest compression” OR “newborn-chest compression” OR “infant cardiopulmonary resuscitation” OR “newborn cardiopulmonary resuscitation”. The following terms were used to search for “simulator” using the “OR” operator: “manikin” OR “mannequin”.

### 2.3. Eligibility Criteria

All studies were independently screened by two authors (CYC and YJC) on the basis of the inclusion criteria: (a) randomized controlled trial (RCT) design, (b) comparison of TT with TF, (c) studies reporting prespecified chest-compression depth and proportion of complete chest recoil, and (d) outcomes with sufficient information for meta-analysis. Studies were excluded if they did not meet the inclusion criteria.

### 2.4. Risk of Bias in Individual Studies

Two authors (CYC and YJC) evaluated the methodological quality of all included studies by using the revised Cochrane risk-of-bias tool for randomized trials (RoB 2) [7]. The third author (MYW) provided consensus or discussion in the case of disagreements. 

### 2.5. Data Extraction

Information included in the studies was extracted by two authors (CYC and YJC), including author names, country, the number and expertise of participants, CPR duration, ventilation protocol, manikin models and placement, and effect estimates. Due to the nature of the crossover RCTs, we extracted continuous outcome data with the following strategy: (1) mean paired difference with 95% confidence interval (CI) was preferred, where available, to (2) mean, standard deviation (SD) of each intervention, and *p*-value from paired t-test, followed by (3) mean, SD of each intervention, and the assumed correlation of the two interventions.

### 2.6. Statistical Analysis

The effect estimate of the outcomes in each study was calculated as mean difference (MD) and standard error (SE) through the different aforementioned data-input strategies. When the “mean, SD of each intervention, and *p*-value from paired t-test” format was chosen, and the upper-bounded *p*-value was reported (e.g., *p* < 0.001), we made a conservative approximation by taking *p* = 0.001 [8]. When the “mean, SD of each intervention, and the assumed correlation of the two interventions” format was chosen, correlation was assumed using the lowest observed correlation among other studies [8]. The summary measurement (either MD or Hedges’ g, where suitable) with the 95% CI was then derived from pooling the effect estimate of each included study using an inverse-variance method with a random-effects model (DerSimonian–Laird estimator [9]). Heterogeneity was assessed by the Cochran Q statistic and quantified with the I^2^ statistic. A priori subgroup analysis was performed to evaluate whether the prespecified factors could account for heterogeneity, including ventilation protocol, country, manikin models and placement, and the expertise of the participants. 

Sensitivity analysis was performed in the following manner to test the robustness of the results. First, influence analysis was performed by omitting one study at a time and reperforming meta-analysis to evaluate if the pooled summary measurement lay within the 95% CI of the overall summary measurement. Second, in outcomes containing studies where the data input involved the assumption of correlation, we replaced the originally assumed correlation (i.e., the lowest observed) with the highest observed correlation among the other studies and zero, and reperformed meta-analysis. Third, a graphical display of study heterogeneity (GOSH) plot was drawn [10], and the potential outlier(s) were identified by three unsupervised-learning algorithms, i.e., k-means clustering [11], density-based spatial clustering of applications with noise (DBSCAN) [12], and Gaussian mixture models [13]. Meta-analysis was reperformed after excluding the potential outlier(s) to test if they had significantly influenced the pooled results. 

Lastly, we evaluated the publication bias in the outcomes from ≥10 studies [14] by a contour-enhanced funnel plot [15]. Egger’s test was used to test for asymmetry [16]. In the case of significant asymmetry, Duval and Tweedie’s trim-and-fill procedure was performed to identify missing studies that should have been plotted [17]. Data input, the computation of the effect size, and the standard error in each included study were performed using Comprehensive Meta-Analysis Version 3 [18]. Further statistical analyses were performed using R version 3.6.0 [19] with “dmetar,” “meta”, and “metafor” packages.

## 3. Results

### 3.1. Study Identification and Selection

**Figure 1:**
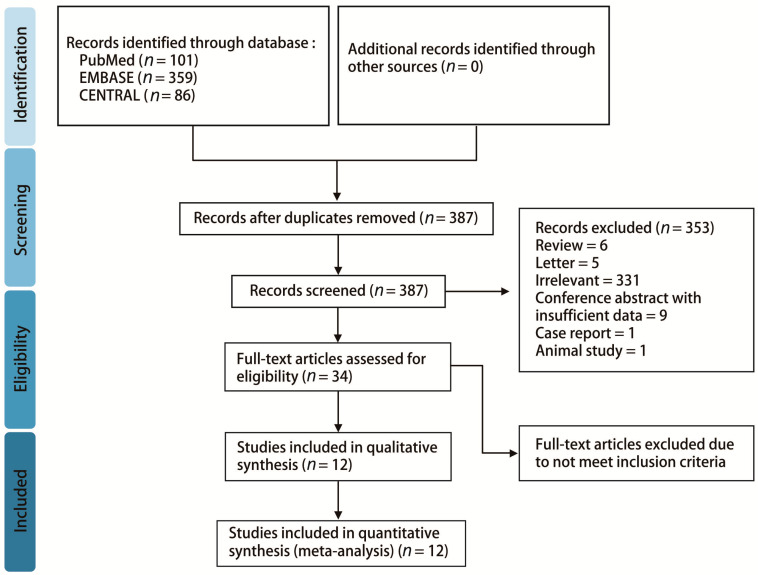
Preferred reporting items for systematic review and meta-analysis (PRISMA) flow diagram.

A total of 546 records were identified from three databases, namely, PubMed (*n* = 101), EMBASE (*n* = 359), and CENTRAL (*n* = 86). After removing 159 duplicates, the remaining studies were screened for eligibility. Of them, 353 were excluded due to being irrelevant, review articles, letters, conference abstracts with insufficient information, case reports, or animal studies. As a result, 34 studies were assessed with full-text review. Twenty-two studies were excluded after full-text review for not meeting the eligibility criteria. Notably, the study by Lee et al. was excluded because they used a compression-to-ventilation ratio of 15:2 [20]. Finally, 12 studies involving 407 participants were included. A detailed PRISMA flow diagram is shown in Figure 1.

### 3.2. Study Characteristics and Quality, and Risk of Bias Assessment

**Figure 2:**
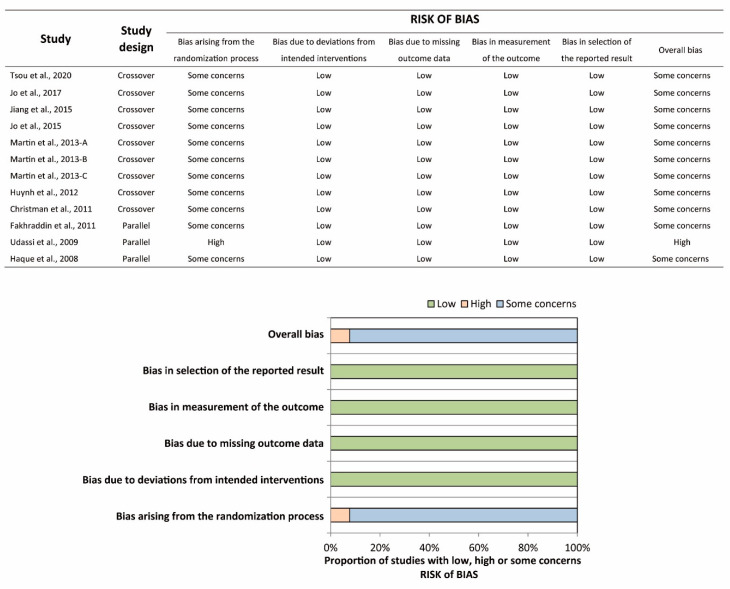
Risk-of-bias summary, and graph of chest-compression depth and proportion of complete chest recoil.

**Figure 3:**
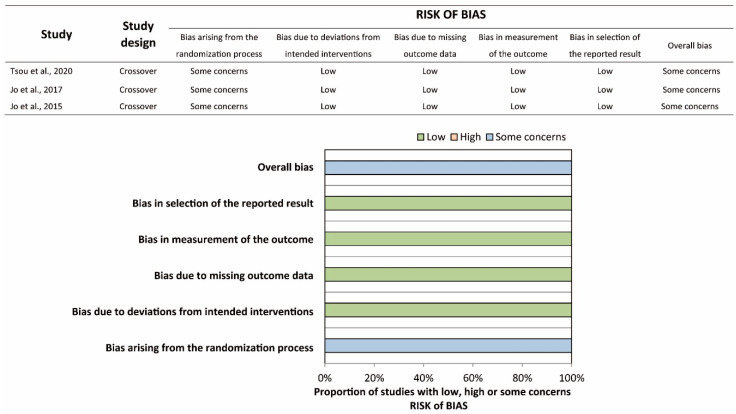
Risk-of-bias summary and graph of proportion of complete chest recoil.

The characteristics of the included studies are shown in Table 1. All included studies were RCTs. In the Haque et al. study [21], a total of 80 participants were randomly allocated to five groups with 16 participants in each group, namely, infant TF, infant TT, child one-hand, child two-hand, and adolescent two-hand groups. Participants in each group were further randomized into two sequences, starting with a compression:ventilation (C:V) ratio of 30:2 or 15:2. We extracted the relevant data in the infant TF and infant TT groups with only a C:V ratio of 30:2. Hence, the Haque et al. study should be regarded as a parallel RCT in the present study. All studies compared the conventional TT technique to the TF technique. Of note, participants were asked to stand at the head position while performing the TT technique (over-the-head TT) in two studies [22,23]. CPR duration ranged from 1 to 5 min. Participants were asked to perform ventilation with a C:V ratio of 30:2 in 7 studies, whereas the others did not require the participants to perform ventilation. Participants were recruited from multiple expertise areas in most of the studies, except for four studies where participants were recruited from a single expertise area [22,23,24]. The risk of bias was assessed for each outcome, and the summary is shown in Figure 2 and Figure 3. Most of the studies were of some concern due to the possible bias arising from the randomization process. Specifically, the generation of the random sequence, and the concealment of the allocation sequence were rarely reported. Moreover, in most of the crossover RCTs, information of baseline imbalances was limited, since the participants’ demographics were reported as a whole instead of as two allocation groups.

### 3.3. Overall Summary Measurement

**Figure 4:**
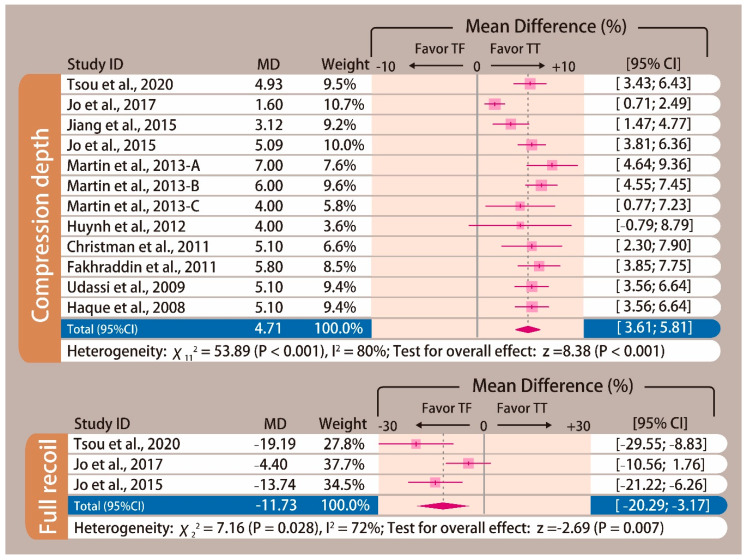
Forest plot of chest-compression depth and proportion of complete chest recoil.

Performing CPR on an infant manikin with the TT technique had significantly deeper chest-compression depth than that of the TF technique (MD: 4.71 mm; 95% CI, 3.61 to 5.81; *p* < 0.001; Figure 4). The proportion of complete chest recoil was achieved more using the TF technique than the TT technique (MD: −11.73%; 95% CI, −20.29 to −3.17; *p* = 0.007; Figure 4).

### 3.4. Subgroup Analysis in Chest-Compression Depth

**Figure 5:**
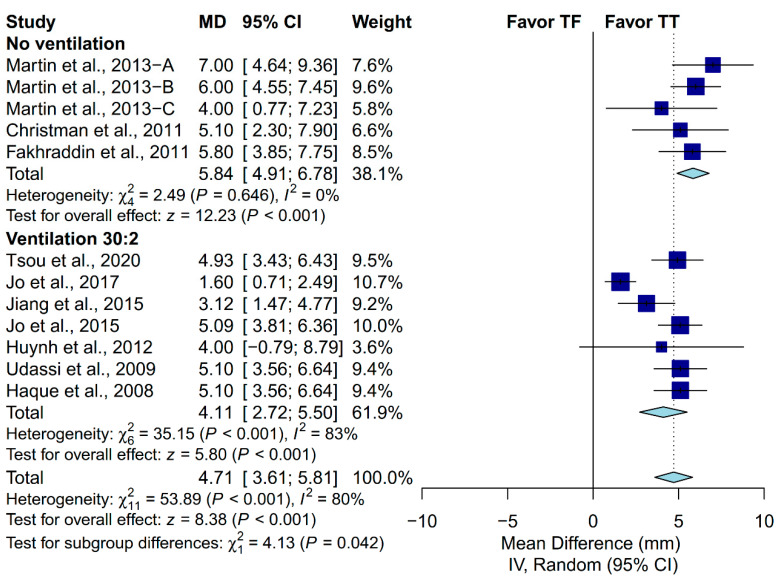
Subgroup analysis of chest-compression depth grouped by ventilation protocol.

**Figure 6:**
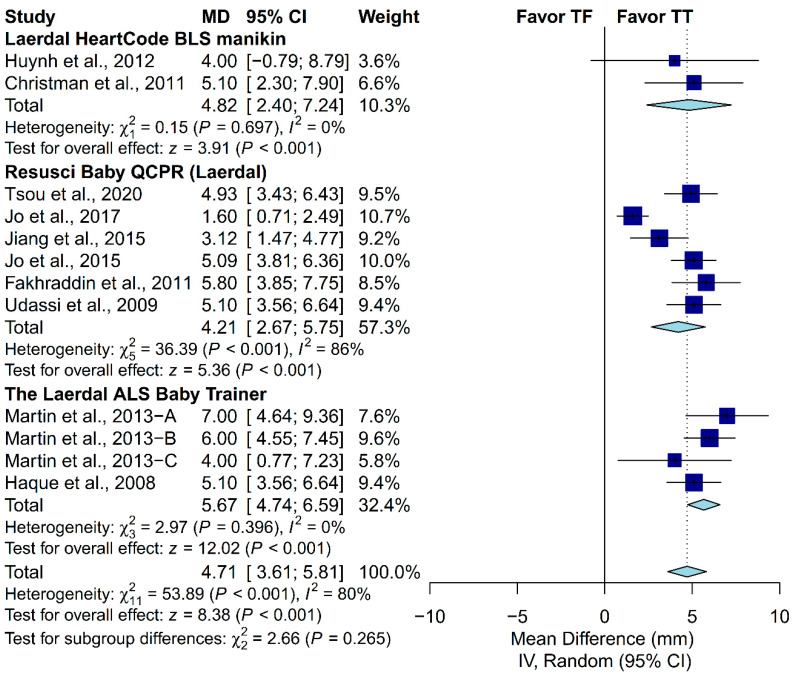
Subgroup analysis of chest-compression depth grouped by manikin model.

**Figure 7:**
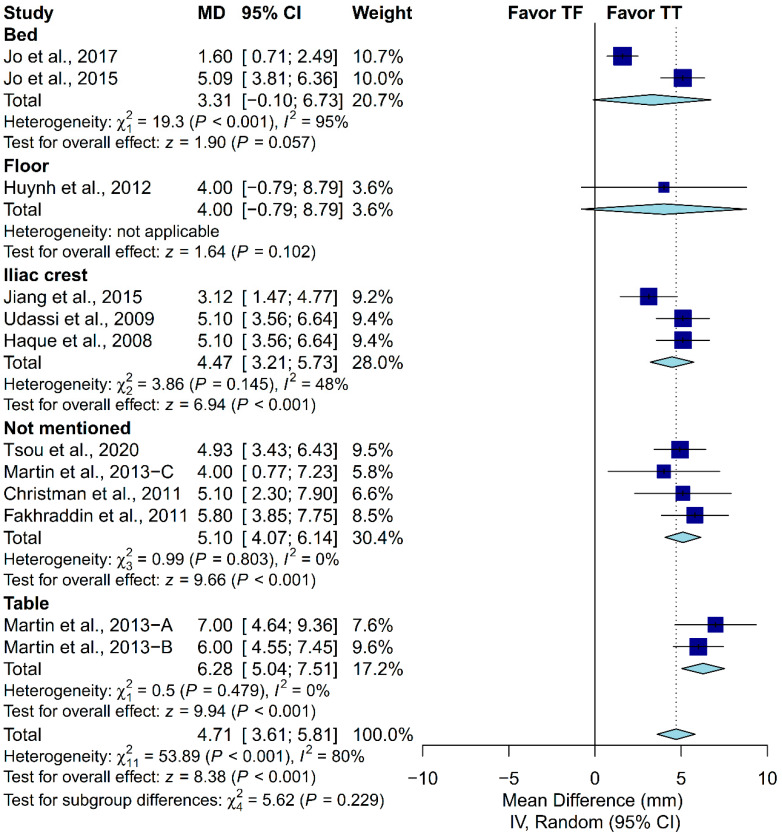
Subgroup analysis of chest-compression depth grouped by manikin placement.

**Figure 8:**
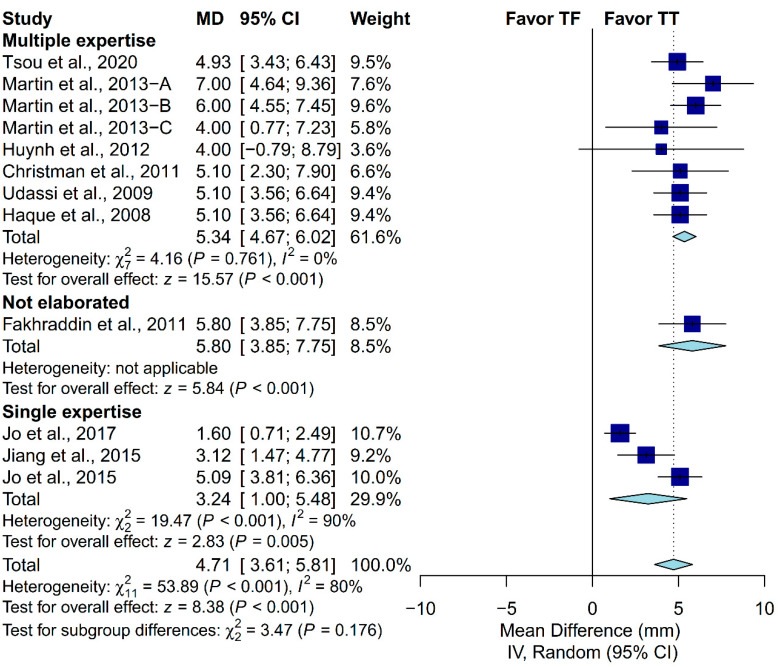
Subgroup analysis of chest-compression depth grouped by participant expertise.

**Figure 9:**
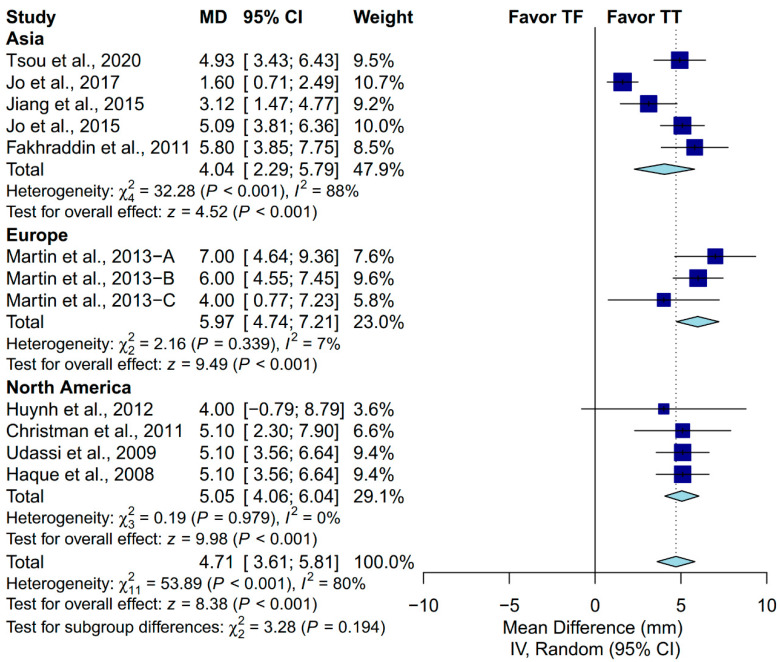
Subgroup analysis of chest-compression depth grouped by locale.

A priori subgroup analysis was conducted to evaluate if the prespecified factors accounted for the heterogeneity observed in chest-compression depth. Subgroup analysis was not conducted in the proportion of complete chest recoil due to the relatively low number of included studies. We found that locale, ventilation protocol, manikin model and placement, and the expertise of the participants could not explain the heterogeneity observed in chest-compression depth (Figure 5, Figure 6, Figure 7, Figure 8 and Figure 9). First, studies conducted in Europe (I^2^ = 7%) and North America (I^2^ = 0%) showed low heterogeneity, but not studies conducted in Asia (I^2^ = 88%). Second, studies in which the participants were not required to perform ventilation showed low heterogeneity (I^2^ = 0%), but not those requiring participants to perform ventilation with a C:V ratio of 30:2 (I^2^ = 83%). Third, studies using the Laerdal HeartCode BLS manikin (I^2^ = 0%) and the Laerdal ALS Baby Trainer (I^2^ = 3%) showed low heterogeneity, but not those using Laerdal Resusci Baby QCPR (I^2^ = 86%). Fourth, studies enrolling participants from multiple expertise areas showed low heterogeneity (I^2^ = 0%), but not those with a single expertise area (I^2^ = 90%). Finally, studies in which the manikin model was placed on the table (I^2^ = 0%) or height adjusted to the iliac crest (I^2^ = 48%) showed low-to-moderate heterogeneity, but not those with the manikin on the bed (I^2^ = 95%).

### 3.5. Sensitivity Analysis in Chest-Compression Depth

**Figure 10:**
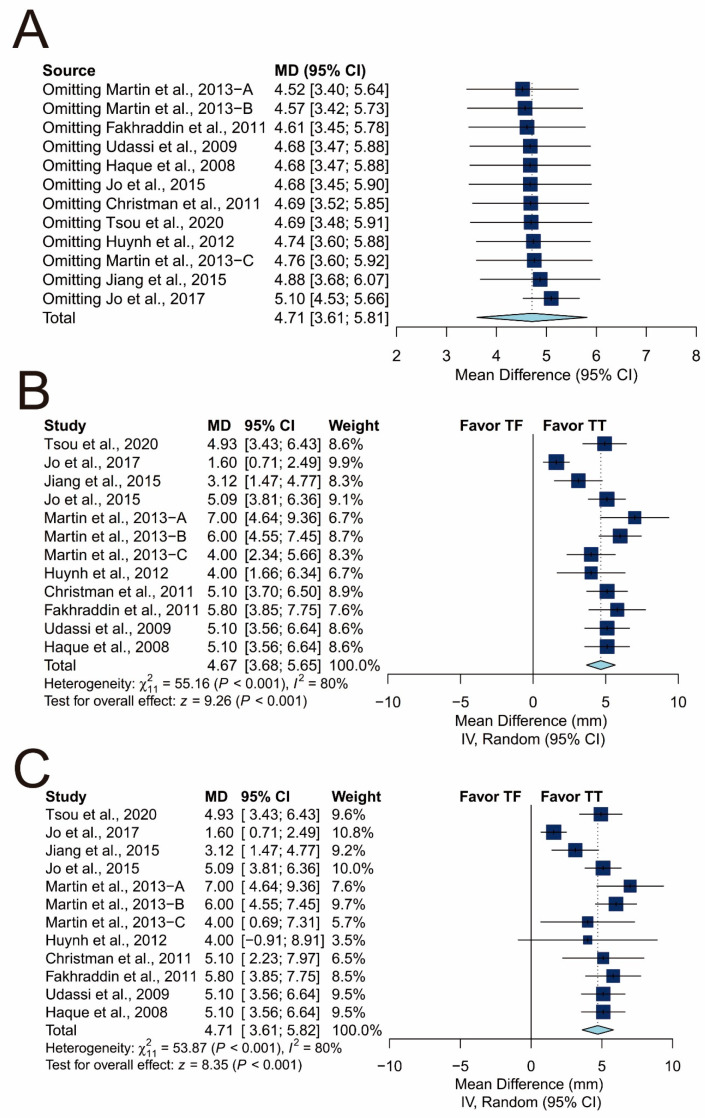
Sensitivity analysis of chest-compression depth. (**A**) Influence analysis; (**B**) forest plot with correlation set as highest observed (0.78); (**C**) forest plot with correlation set as 0.

**Figure 11:**
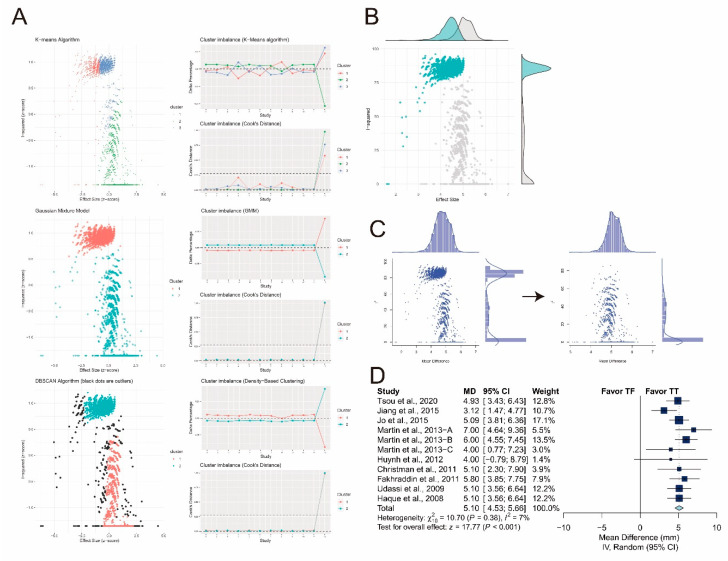
(**A**) Potential outliers identified by three unsupervised-learning algorithms; (**B**) graphical display of study heterogeneity (GOSH) plots with corresponding subsets including potential outliers colored in cyan; (**C**) (left) original GOSH plot; (right) GOSH plot after excluding potential outliers; (**D**) forest plot after excluding potential outliers.

First, influence analysis revealed that all the pooled estimates after omitting one study at a time still lay within the 95% confidence interval of the overall estimate (Figure 10A). Second, the lowest observed correlation of the TT and TF techniques among the other studies, which is 0.05, was assumed for three studies [28,29,30] due to the chosen data-input strategy described in Section 2.5 and Section 2.6. Here, we replaced the original correlation with the highest observed, which was 0.78, and zero, and reperformed meta-analysis. The overall estimate remained significant after correlation was replaced with the highest observed one (MD: 4.67 mm; 95% CI: 3.68 to 5.65; *p* < 0.001; Figure 10B) and zero (MD: 4.71 mm; 95% CI: 3.61 to 5.82; *p* < 0.001; Figure 10C). Third, the GOSH plot showed at least two subclusters (Figure 11A). Three unsupervised-learning algorithms identified one potential outlier [22] (Figure 11A). The corresponding subsets, including the potential outlier, are shown in Figure 11B. After excluding the potential outlier, the GOSH plot became relatively homogenous (Figure 11C). We reperformed meta-analysis after excluding the potential outliers, and the pooled estimate remained significant (MD: 5.10 mm; 95% CI: 4.53 to 5.66; *p* < 0.001; Figure 11D) with low heterogeneity (I^2^ = 7%).

### 3.6. Influence Analysis in Complete Chest Recoil 

**Figure 12:**
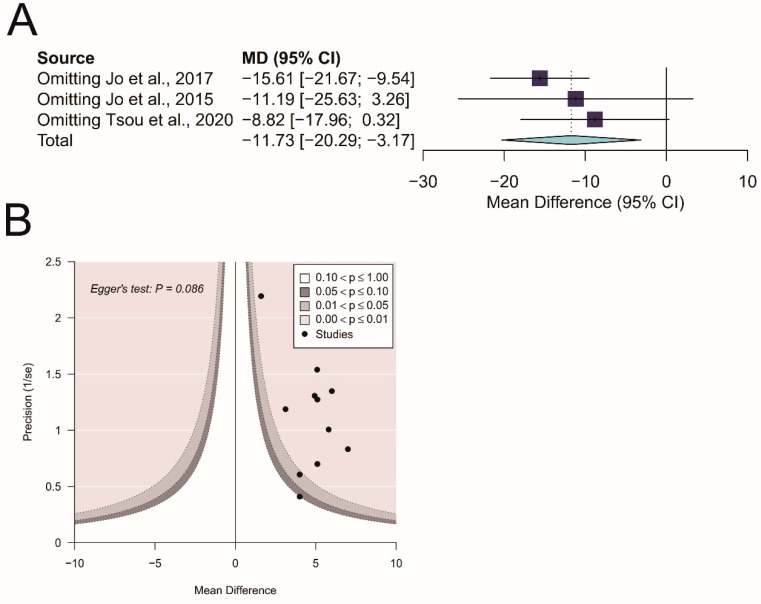
(**A**) Influence analysis of complete chest recoil; (**B**) contour-enhanced funnel plot of chest-compression depth (Egger’s test for asymmetry: *p* = 0.086).

Influence analysis was performed for complete chest recoil. Results revealed that all pooled estimates after omitting one study at a time still lay within the 95% confidence interval of the overall estimate (Figure 12A).

### 3.7. Publication Bias

A contour-enhanced funnel plot revealed no asymmetry in chest-compression depth (*p* = 0.086 by Egger’s test; Figure 12B).

## 4. Discussion

Our results showed that the TT technique generated significantly better compression depth than the TF technique did. This was similar to previous studies. In subgroup analysis, the compression depth using the TT technique was deeper whether or not ventilation was performed. The advantage of the TT technique in compression depth remained in different rescuer populations. In addition, the TT technique was superior to the TF technique when infants were placed in different settings, including the floor, at the height of the iliac crest, and a table. Several reasons may explain these results. First, during chest compression, the TF technique is relatively unsteady due to the absence of the fixed rescuer’s elbow. Second, the TF technique has less contact area for the stabilization of chest compression. Third, the TF technique is not associated with a natural position, and may thus be more energy-consuming and result in fatigue during CPR. Finally, the TF technique has high compression pressure only at the fingertips, which may easily be misplaced with fatigued rescuers. In the Tsou et al. study [25], the mean compression force of the TT technique was significantly higher than that of the TF technique, and the decrease of compression-force delivery during CPR was also significant in the TF technique. With respect to ergonomics, the TT technique is much superior to the TF technique. 

In chest-recoil analysis, the TF technique was associated with a higher proportion of complete chest recoil than that of the TT technique. Complete chest recoil is an important factor affecting coronary blood supply and arterial blood pressure. A higher proportion of complete chest recoil may increase venous return and stroke volume, and improve mean arterial pressure and coronary blood supply. Several reasons may explain the superiority of the TF technique to the TT technique in terms of complete chest recoil. First, the limited range of motion at the interphalangeal and metacarpophalangeal joints of the thumbs may restrain the chest wall from complete recoil. Second, in the TT technique, the circumferential placement of the hands may lead to restriction and jeopardize complete chest recoil. As a result, we inferred that the TF technique may generate higher mean arterial pressure and deliver more coronary blood supply than the TT technique on the basis of the present study. Interestingly, several manikin and animal studies evaluated hemodynamics during CPR, and revealed that the TT technique produced higher systolic and diastolic arterial pressure compared to the TF technique [33,34,35,36]. In the Dorfsman et al. study [35], the TT technique produced higher systolic blood pressure (marginal means: 68.9 vs. 44.8), diastolic blood pressure (marginal means: 17.6 vs. 12.5), mean arterial pressure (marginal means: 35.3 vs. 23.3), and pulse pressure (marginal means: 51.4 vs. 32.2) compared to the TF technique. The proposed mechanism for higher pressure in the TT technique was the combination of thoracic- and cardiac-pump mechanisms. The circumferential application of force provided greater thoracic pressure than the TF technique did. Although the circumferential placement of the hands may lead to restriction and jeopardize complete chest recoil, we speculated that the effect of compression depth may play a more important role than that of complete chest recoil in HP-CPR.

Despite the obvious advantage of the TT technique in generating greater compression depth, we could not make a solid and strong recommendation that the TT technique be routinely used for infant CPR in the setting of a lone rescuer due to the limitation of achieving complete chest recoil. Nevertheless, several modified TT techniques were proposed to overcome this disadvantage. For instance, Smereka and colleagues came up with the new two-thumb technique (nTTT) that required the rescuers to place two thumbs directed at the angle of 90° to the chest wall while closing the fingers of both hands in a fist [37]. The nTTT resulted in greater compression depth and more complete chest recoil than the TF technique did. In contrast, the TT technique generated greater compression depth at the cost of significantly incomplete chest recoil. The newly proposed and modified TT technique introduced by Smereka et al. is promising. Further large-scale RCTs are warranted to confirm the efficacy of this technique.

The present study differed from a recent study published by Millin et al. in several ways [38]. First, in the study by Millin et al., they reported better and more consistent chest-compression depth with the TT technique, which is similar to our findings. However, we additionally evaluated complete chest recoil that is a crucial component known to contribute to HQ-CPR [39]. Second, one more eligible study was identified and included in the present study [25]. Third, we adopted a GOSH plot and unsupervised-learning algorithms to aid in exploring potential outliers contributing to the substantial heterogeneity observed in our results. However, there were several limitations in this study. First, although coronary perfusion pressure (CPP) is the most direct indicator of chest-compression quality in terms of the heart, no studies to date have reported this outcome. Therefore, we summarized indirect parameters, such as diastolic blood pressure and mean arterial pressure, to reflect CPP. Second, all included studies evaluated the CPR quality of the two techniques on a manikin model. Characteristics of chest stiffness and resistance may be different from those in real human beings, and may cause different results. Third, the sample size was relatively small. Lastly, most of the enrolled participants were emergency medical technicians, registered nurses, or physicians. Our results may not apply to other rescuers, especially uneducated bystanders.

## 5. Conclusions

In the setting of a lone rescuer, the TT technique is superior to the TF technique in terms of greater compression depth. However, the TT technique is inferior to the TF technique in terms of complete chest recoil. For the time being, recommendations that the TT technique be used routinely for infant CPR with a lone rescuer cannot be made. Nonetheless, the modified nTTT is promising in that it generates greater compression depth while preserving complete chest recoil. Further large-scaled RCTs are warranted to investigate the efficacy of nTTT.

## Figures and Tables

**Table 1:** Detailed characteristics of included studies.

Study	Country	n	Comparison	CPR Duration (min)	Ventilation *	Manikin	Manikin Placement	Participants
Tsou et al., 2020 [25]	Taiwan	42	TT vs. TF	2	30:2	Resusci Baby QCPR (Laerdal)	Not mentioned	EMT, RN
Jo et al., 2017 [22]	Korea	48	OTTT vs. TF	2	30:2	Resusci Baby QCPR (Laerdal)	Bed	Medical students
Jiang et al., 2015 [24]	China	27	TT vs. TF	5	30:2	Resusci Baby QCPR (Laerdal)	Iliac crest	Physicians
Jo et al., 2015 [23]	Korea	46	OTTT vs. TF	2	30:2	Resusci Baby QCPR (Laerdal)	Bed	RN
Martin et al., 2013-A [26]	UK	22	TT vs. TF	2	No	The Laerdal ALS Baby Trainer	Table	Physicians, RN, resuscitation officers
Martin et al., 2013-B [27]	UK	40	TT vs. TF	1.5	No	Laerdal ALS Baby Trainer	Table	Resuscitation officer, physicians, RN, operating-room practitioner, paramedics
Martin et al., 2013-C [28]	UK	35	TT vs. TF	1	No	Laerdal ALS Baby Trainer	Not mentioned	Resuscitation officers, physicians, RN
Huynh et al., 2012 [29]	USA	18	TT vs. TF	2	30:2	Laerdal HeartCode BLS manikin	Floor	RN, NP, physicians
Christman et al., 2011 [30]	USA	25	TT vs. TF	1	No	Laerdal HeartCode BLS manikin	Not mentioned	Physicians, RN
Fakhraddin et al., 2011 [31]	Japan	40	TT vs. TF	5	No	Resusci Baby QCPR (Laerdal)	Not mentioned	PALS providers
Udassi et al., 2009 [32]	USA	32	TT vs. TF	5	30:2	Resusci Baby QCPR (Laerdal)	Iliac crest	RN, medical student, physicians, faculty, others
Haque et al., 2008 [21]	USA	32	TT vs. TF	5	30:2	Laerdal ALS Baby Trainer	Iliac crest	Faculty, physicians, RN, medical/nursing students, RT, OT

* Ventilation is presented as either “No” or compression:ventilation ratio; *n*: patient number; TT: two-thumb technique; OTTT: over-the-head two-thumb technique; TF: two-finger technique; CPR: cardiopulmonary resuscitation; EMT: emergency medical technician, RN: registered nurse; NP: nurse practitioner; PALS: pediatric advanced life support; RT: respiratory therapist; OT: occupational therapist.
